# State Union Density Effects on Workers’ Support for Reducing Income Inequality, 1973-2016

**DOI:** 10.1177/23294965221089914

**Published:** 2022-05-26

**Authors:** Shawn Perron

**Affiliations:** 1 University of Toronto, Toronto, ON, Canada

**Keywords:** inequality, poverty and mobility, political sociology, organizations, occupations, and work

## Abstract

Research often borrows on common yet somewhat unsubstantiated beliefs that unions influence inequality attitudes among unionized and nonunionized workers. This paper draws on inequality attitude data from the General Social Survey and state-level union data from the Current Population Survey and County Business Patterns between 1973 and 2016 to test this hypothesis. Linear probability, fixed-effects, and marginal structural models estimate that a large increase in state union density moderately increases workers’ support for reducing income inequality by three to 12 percentage points. Findings lend some empirical support for the capacity of unions to influence redistributive policy and market attitudes.

## Introduction

The distribution of personal income in the US has become substantially more unequal since the early 1980s, reversing a general trend of declining inequality dating back to the 1930s. During the 1980s and early 1990s, incomes in the lower half of the distribution stagnated and then declined, while incomes at the top of the distribution increased. During the late 1990s and 2000s, incomes in the lower part of the distribution ceased declining but did not rebound from the losses of previous decades, while top incomes continued their ascent (Morris and Western 1999; Piketty and Saez 2006; McCall and Percheski 2010). Several mechanisms have contributed to this trend, including financialization; increasing returns on capital; changes in class structure; declines in regulatory institutions such as unions, the minimum-wage, and taxation; and skill-biased technological change (Morris and Western 1999; Card and DiNardo 2002; McLanahan and Percheski 2008; Western and Rosenfeld 2011; Lin and Tomaskovic-Devey 2013; Piketty 2014; Wodtke 2016). The recent increase in income inequality is alarming because greater inequality is associated with a variety of social, political, and economic problems, such as lower levels of health and wellbeing; higher levels of social and political unrest; and slower economic growth (Dabla-Norris et al. 2015; Ostry, Berg, and Tsangarides 2014; Pickett and Wilkinson 2015; Patel et al. 2018). Several world leaders including the US President and members of the International Monetary Fund and World Economic Forum have identified income inequality as one of the defining challenges of our time (Lagarde 2013; Obama 2014).

While academics and some politicians recognize income inequality as an important social problem, average workers attitudes towards income inequality are also important for understanding inequality trends (Western and Rosenfeld 2011; Lamont, Beljean and Clair 2014). Studies measure inequality attitudes from a variety of survey questions including support for income redistribution; support for tax progressivity or other welfare policy; and whether the income gap is too large (Meltzer and Richard 1981; Milanovic 2000; McCall 2013; VanHeuvelen 2016; Macdonald 2019). While each question is unique, agreement with each suggests some support for reducing income inequality (hereafter shortened to SFRI). Scholars argue that SFRI may constrain and enable market behavior and political decisions, such as compensation practices and welfare policies that influence income inequality (Western and Rosenfeld 2011; Lamont, Beljean and Clair 2014). The median-voter perspective argues that SFRI increases as income inequality rises, because more individuals will stand to benefit from redistributive policy (Meltzer and Richard 1981; Milanovic 2000; VanHeuvelen 2016). Others argue that SFRI is sensitive to media narratives that link income inequality to inequitable economic growth, barriers to social mobility, or other harms to the “American Dream” (Lamont 1992; McCall 2013). For example, mainstream media coverage of the 2008 economic recession and 2011 Occupy Wall Street and sister movements helped disseminate and bring a sense of urgency to inequality trends, increasing SFRI for some individuals (McCall 2013). Yet, such concern about income inequality is not widely shared; many believe social mobility is possible despite rising income inequality and that high incomes are a result of hard work and talent rather than networks or inherited privilege (Sawhill and Morton 2007).

In addition to the media and other institutions, scholars often assume that unions influence inequality narratives and attitudes (Svallfors 2006; Kumlin and Svallfors 2008; Western and Rosenfeld 2011). Historically, successful union campaigns associated with Caesar Chavez and Walter Reuther relied on fostering egalitarian values and scrutinizing the very rich (Dray 2011; Western and Rosenfeld 2011). Others argue that unions help establish local pay norms (such as compensation that is “too high”) and social sanctions against those who violate them (Korpi 1983; Korpi 1985; Svallfors 2006; Kumlin and Svallfors 2008; Western and Rosenfeld 2011). Unions may also help disseminate information from politicians, the news, and other media to help explain the relationship between inequality, redistributive policies, and its effects on the economy. Nonunionized workers also access these attitudes through union newspapers, speeches at protests, networking events, and informal communication with their unionized friends and family (Black 2005; Mollona 2009; Western and Rosenfeld 2011; McAlevey 2016; Mosimann and Pontusson 2017). The capacity to harness local attitudes is also used by moral economy and Power Resource scholars, in part, to explain how unions successfully restrict compensation practices and lobby for welfare policies, like increasing the minimum wage (Korpi 1983; Korpi 1985; Esping-Andersen 1990; Svallfors 2006; Western and Rosenfeld 2011). Given the decline in union membership from about 25 to 10% of American workers since the 1980s, contemporary Americans may be less persuaded by (or lack important information for understanding) the harmful effects of rising income inequality.

Beliefs that unions successfully foster(ed) attitudes among workers and their communities, despite being common, lack strong substantive evidence. From this hypothesis, scholars may expect that countries and regions with a higher proportion of union members must also have higher SFRI. However, existing research shows that union density effects on SFRI are largely mixed. State and country comparative studies show positive union density effects on average support for redistribution, while others show negative or mixed union density effects on other indicators of SFRI after controlling for demographic covariates and individual union membership (Kumlin and Svallfors 2008; Franko 2016; Mosimann and Pontusson 2017; Yang and Kwon 2019; Macdonald 2019). Documenting this relationship is challenging because individual survey questions often fail to capture the nuance of inequality attitudes and are included in few survey waves.

This paper tests the long-lasting assumption that union density increases SFRI within communities by drawing on state-level union membership and union local data from the Current Population Survey (CPS) and County Business Patterns (CBP) between 1973 and 2016. Union density estimates are matched with seven inequality-related attitudinal questions asked of individuals across multiple waves of the General Social Survey (GSS). Results from Linear Probability Models (LPMs) and Fixed-Effects models show that after controlling for individual and state covariates, a large increase in state union density (23% union membership or 52 locals per 500,000 population) moderately increases workers’ SFRI (by three to 12 percentage points). Testing this hypothesis is important because moral economy and Power Resource scholars, among others, argue that unions’ capacity to foster attitudes helps explain their effects on market practices and welfare policy (Korpi 1983; Korpi 1985; Esping-Andersen 1990; Svallfors 2006; Western and Rosenfeld 2011). Until now, this assumption has been largely speculative, leaving scholars with limited evidence for evaluating whether unions influence markets and policy through culture or some other mechanism, such as closed-door negotiations with employers and politicians on regulatory boards and other institutions. Findings imply that the mediating role of culture should be seriously considered when explaining well-known union density effects on rising income inequality, welfare policy, and other market and policy characteristics. Findings also have implications for the narrative approach to inequality attitudes, suggesting that union speeches, newsletters, and other activity successfully raise concern for income inequality. Explanations of SFRI should seriously consider the role of unions and narratives alongside other mechanisms emphasized by the median-voter perspective.

## When, Where, and Why Income Inequality Should be Reduced

Scholars argue that SFRI—often measured as support for income redistribution policies—helps explain why some countries, like those in Scandinavia, have comparatively strong welfare programs (Meltzer and Richard 1981). SFRI is higher when workers (1) anticipate rewards from redistributive policy and/or (2) believe inequality harms the economy. The first mechanism—referred to as the median-voter model—hypothesizes that SFRI increases when inequality reaches the extent that even median-income earners can expect rewards from more progressive taxation or other redistributive income policies (Meltzer and Richard 1981; Milanovic 2000; VanHeuvelen 2016). Others draw on profit-assessing behavior to explain variation in policy preferences between groups. For example, richer workers are less likely to support redistribution than poorer workers because they are less likely to benefit from redistribution (Meltzer and Richard 1981; Milanovic 2000; VanHeuvelen 2016). Older Americans are less supportive of universal healthcare because it risks cuts to their Medicare (Ashok, Kuziemko and Washington 2015). Welfare spending targeting racialized or income groups likewise receive less broad support because fewer expect subsequent rewards (Korpi and Palme 1998; Schmidt and Spies 2014).

The median-voter model is comparable to the thermostatic model of inequality attitudes, which hypothesizes that policy preferences respond dynamically to policy spending. Individuals desire to “turn down the heat” on redistributive policy as related spending increases and “turn up the heat” as it declines (Wlezien 1995; Pacheco 2013). Both the thermostatic and median-voter models suggest that support for redistribution is a function of trends in income inequality and related policy spending. They also both assume that individuals are relatively well informed of income inequality trends and related policy, which is not always well supported (Bartels 2005; Osberg and Smeeding 2006; Kenworthy and McCall 2008; Bartels 2010; Norton and Ariely 2011; McCall 2013).

In addition to inequality trends, inequality attitudes are influenced by the “cultural supply side.” Lamont (1992:7) defines the cultural supply side as the “cultural repertoires, traditions, and narratives that individuals have access to.” McCall (2013) draws on this perspective to analyze how narratives about inequality, such as those shared by the news, politics, communities, and other organizations influence inequality attitudes. For example, trends in public opinion about income inequality between the 1980s and 2000s resemble trends in media coverage of income inequality during this period. SFRI also increases when individuals believe that (1) help from other people is more important than hard work for getting ahead, (2) the rich earn above a “fair” wage, (3) inequality harms economic growth, and (4) egalitarianism is good for the country (McCall 2013). In other words, individuals are more likely to show concern for growing income inequality when narratives associate the “very rich” with limited opportunities or slower economic growth. Narratives about the harm or benefits of income inequality may present more alluring and moving information to remember and share than inequality trends.

While many institutions influence inequality narratives, the roles of political parties and unions are often cited. Election seasons see heightened media coverage of economic and policy debates that frame income inequality ambiguously, positively, or negatively. Republican organizations often mobilize for tax-breaks, arguing that they help the very rich create jobs and inequality may motivate innovation and entrepreneurship. Barack Obama, on the other hand, identified inequality as the “the defining issue of our time” and a barrier against social mobility (Obama 2014). Research suggests that such narratives are moderately effective at influencing attitudes. “Strong Democrats” are more supportive of income redistribution than “strong Republicans” on average (McCall 2013). Historically Democratic regions in the US also have more support for egalitarian and collectivist attitudes than historically Republican regions (Hogler, Hunt and Weiler 2015).

Scholars also argue that unions may influence regional inequality attitudes. Historically successful union leaders like Cesar Chavez and Walter Reuther are well known examples of community organizers that lobbied for redistribution, civil rights, and egalitarian ideals among workers and their communities (Dray 2011). Contemporary union organizing strategies also emphasize “whole worker organizing” and “community organizing” through grassroot networks that spread information about economic issues and reform across neighborhoods (Black 2005; Mollona 2009; McAlevey 2016). Unions also communicate these ideas to nonunionized workers through newsletters, speeches at public events, and the informal networks of union members. Such community ties, unionists argue, are essential for mobilizing votes and protests that pressure strong employers and policy makers (McAlevey 2016). Scholars also argue that unions help disseminate economic information about inequality, identifying policy solutions, and fostering income norms that construe what is “too much” or “too little” compensation (Korpi 1983; Freeman and Medoff 1984; Korpi 1985; Svallfors 2006; Kumlin and Svallfors 2008; Western and Rosenfeld 2011; Mosimann and Pontusson 2017; Macdonald 2021).

SFRI may also function as a mechanism of union density, explaining in part how unions influence welfare regimes and inequality trends. Countries with stronger welfare regimes/policies tend to have more union members and US interstate regions and industries with more deunionization have more wage dispersion (Korpi 1985; Bradley et al. 2003; Western and Rosenfeld 2011). Power Resource Theory suggests that unions increase welfare spending in part by fostering strong “negative attitudes” against individuals who increase inequality or make welfare cuts, allowing workers to “punish” or “reward” those in power (Stephens 1979; Korpi 1983; Korpi 1985; Esping-Andersen 1990; Jacobs and Dirlam 2016; VanHeuvelen 2020). Similarly, the moral economy perspective argues that unions reduce income inequality in part by fostering strong egalitarian pay norms (Svallfors 2006; Western and Rosenfeld 2011). Both perspectives suggest that when there is a strong union presence, employers and politicians may be more cautious of violating norms towards appropriate compensation practices and levels of income inequality. These perspectives build on scholarly traditions inspired by E.P Thompson and Karl Polanyi, who argue that workers with sufficient organizing power are capable of "embedding" market practices in local norms and attitudes (Polanyi 1944; Thompson 1971). Alongside other important strategies available to unions, such as membership on regulatory labor boards, bargaining with employers, and lobbying political organizations, these perspectives refer to the capacity to harness local attitudes as valuable for understanding how unions successfully influence policy and market practices.

While many reasonably assume that unions influence inequality attitudes, given the history and aims of unions, empirical support is somewhat inconsistent. Individual union members are more supportive of redistribution on average (Checchi, Visser and Van De Werfhorst 2010; VanHeuvelen 2016; Mosimann and Pontusson 2017) and “social democratic” countries with more union members tend to have higher support for redistribution (Svallfors 1997; Svallfors 2012; Mosimann and Pontusson 2017). Yet, other studies document negative regional union density effects on support for redistribution after controlling for individual union membership (Mosimann and Pontusson 2017; Yang and Kwon 2019). In the US, some studies suggest that union density is positively associated with support for “liberal ideology” and education spending yet not welfare spending generally (Franko 2016) while other studies show that union density is positively associated with support for welfare spending (Macdonald 2019). These studies—stemming from different fields and methodological backgrounds—show a lack of consensus on union density effects across different indicators of SFRI.

Estimates of union density effects on SFRI are often complicated by contextual factors and the period of analysis. Unions experienced major declines in membership between the 1980s and 1990s, while income inequality was steadily increasing. While union decline occurred in several countries, this trend was particularly sharp in the US and has not recovered. The US case is characterized by the Regan Administration’s “union busting” in the 1980s; a famous 1981 case where Regan declared striking airline traffic control workers (PATCO) in violation of the law and at risk losing their jobs set a standard for firms across the country to avoid union bargaining and strikes by prosecuting and/or replacing workers (Dray 2011). This period also observed the collapse of manufacturing industries—referred to as deindustrialization—which made up a significant portion of union members. Throughout the 20^th^ century, US unions also shifted away from social unionism—emphasizing large membership size and social movements—and towards business unionism that focuses on bargaining within firms. A series of acts in the 1930s and 40s restricted union bargaining to firm-related issues, such as wages, hours, and work conditions. Organizations such as the Federal Mediation and Conciliation Service were also introduced to regulate and prohibit some strike activity and negotiating tactics (including secondary strikes sit-down strikes, sympathy strikes, and sympathy boycotts). Scholars argue that business unionism limits the capacity of US unions to organize around working class issues, lobby for related policy change, and reduce inequality (Tomlins 1985; Rubin 1988; Mollona 2009; Rosenfeld 2014). With union resources significantly hampered, it is plausible that unions no longer widely influence attitudes (and SFRI) as they have in the past. Measures of state-level SFRI with notable missing data prior to 1980 may lack important data for estimating unions’ cultural effects.

Documenting a relationship between union density and SFRI is also complicated by methodological issues related to isolating union effects and measuring inequality attitudes. Union density is a product of multiple economic characteristics that may also influence attitudes, such as Right to Work laws, political ideologies, unemployment, economic growth, welfare practices, cultural norms, and other characteristics (Mason and Bain 1993; Tope and Jacobs 2009; Hogler, Hunt and Weiler 2015). Clearly isolating union density effects from these other, often unobserved characteristics is methodologically complex. Some studies introduce fixed-effects models to control for unobserved regional characteristics (Mosimann and Pontusson 2017; Yang and Kwon 2019), yet data on both union membership and attitudes is usually only asked of a few waves that do not allow for much variation within regions. On the other hand, the nuance of inequality attitudes complicates measures of SFRI. Some questions directly refer to inequality as harmful while others are more ambiguous, and studies suggest that such framings influence the popularity of different answers and their relationship with union density (McCall 2013; Franko 2016). In sum, scholars currently lack reliable evidence for thoroughly unpacking and documenting the relationship between SFRI and union density.

## Data

I test the hypothesis that union density increases SFRI by using data on inequality attitudes from the GSS matched with state-level data on union membership, demographics, and other regional characteristics from the CPS and other data. The GSS is an omnibus national survey based on a series of independent nationally representative samples of the adult population, using a multistage area probability design. The survey was conducted annually from 1972 to 1994 (except in 1979, 1981 and 1992) and biannually thereafter. As an omnibus national survey, the GSS collects information on a range of topics, including attitudes related to wages, taxes, and inequality. Core questions in the GSS are asked of all respondents in every wave of the survey, while other items are asked of only a random subset of respondents or are only included in the survey periodically. The 1972-2016 independent cross-sections contain information on about 60,000 respondents.

While the GSS includes many inequality-related questions, some are asked in few waves and of few respondents. I focus on questions regularly included in the survey and asked of sufficiently large samples between 1972 and 2016 to precisely measure SFRI over time. This includes seven questions:

Differences in income in America are too large
Inequality continues to exist because it benefits the rich and powerful
Large differences in income are not necessary for America’s prosperity
Government should reduce income differences between the rich and the poor
Government should do everything possible to improve the standard of living for all poor Americans
Taxes are much too low for the rich
Taxes are much too high for the poor

Each question uses a likert (agree-disagree) response scale, with agreement in each case uniquely capturing SFRI and related narratives. Questions one, two, and three refer to inequality directly and whether it is “too large” or harmful to the economy yet do not directly imply whether or how inequality should be reduced. Questions four and five capture beliefs that the government should do something to reduce inequality yet are vague on the remedy. Questions six and seven refer more specifically to taxation, and only indirectly to inequality. To simplify responses, answers above the midpoint (typically “agree” and “strongly agree”) are coded as one, denoting agreement that income inequality is too large and/or should be reduced, and zero otherwise. I also run parallel analyses using the full likert scales that show similar results (available upon request), yet the binary operationalization is selected because it accurately measures "support" for reducing income inequality and is easier to interpret.

I also construct individual level covariates from the GSS. These include the respondent’s age, sex, race, marital status, education, employment status, and union membership. Age is coded as a continuous variable. Gender is dummy coded as one for female and zero for male. Race is dummy coded as one for non-white and zero for white. Marital status is dummy coded as one for not currently married (divorced, widowed, or never married) and zero for currently married. Education is coded as a series of dummy variables, with one (and zero otherwise) in each case representing whether the respondent’s highest education is less than high school, high school, one year or more of college, or one year or more of university. Employment status is dummy coded as one for employee (part-time or full-time) and zero for self-employed. Individual union membership is dummy coded as one for union member and zero for not a union member.

While the GSS includes attitudinal and demographic data, it is not well optimized for accurate measures of union density and other state characteristics. However, the GSS asks respondents for their state of residence that allow researchers to assign state estimates from other data to individuals (with their attitudes) in the GSS. For example, CBP data includes industry information on US businesses at the county level from 1964 onward. These data are compiled by the Census Bureau’s Business Register and incorporates information from business surveys, quarterly and annual Federal Income and payroll tax records, and other Departmental and Federal statistics and administrative programs. I divide the number of “labor union” industries (recorded since 1974) by the resident population in each state and year and estimate union locals per 500,000 resident population. Union locals per 500,000 people is then assigned to individuals in the GSS with the matching state and year.

Estimates of state characteristics also come from the CPS; a monthly labor force survey conducted by the US Census Bureau. The CPS uses representative samples of about 60,000 households, selected to ensure that reliable estimates of key labor force characteristics can be obtained for all states and most metropolitan areas. The survey has asked about employment status, household demographics, and other labor force characteristics, such as union membership and wages since 1973. Extracts are compiled by the National Bureau of Economic Research (NBER). I estimate the proportion of workers that are young (defined as younger than 30), female, non-white, not currently married, without postsecondary education (i.e., 12 and fewer years of completed schooling), not self-employed (employees), and union members (another measure of union density). Information on union membership is missing in 1983 because it was omitted from the survey. These estimates draw on the May extracts of the CPS between 1973 and 1982 and the merged outgoing rotation group (MORG) from 1983 onward.

I estimate state wage inequality from the variance of log wages in CPS, following existing methods (Western and Rosenfeld 2011). Wage data is drawn from the May between 1973 and 1978 and from the MORG afterwards. Imputed wages are excluded from the sample because they bias regression coefficients for non-matching criteria and increase residual wage variance (Hirsch and Schumacher 2004). I calculate wages by dividing weekly earnings by hours worked per week at the respondent’s main job. Wages for hourly workers are replaced with the straight-cut hourly wage series if this value is higher than values calculated from weekly earnings. Wages are top coded in the survey, masking the highest values. I approximate top wages by multiplying top-wage-codes by 1.3. Finally, wages are adjusted for inflation and converted to their log value, which I use to estimate the variance for each state and year.

I also estimate state characteristics from the Correlates of State Policy Project (CSPP), an initiative published by the Institute for Public Policy and Social Research and organized by a group of scholars to compile yearly state estimates of political, social, and economic factors that may influence policy differences (Jordan and Grossmann 2020). These data include the political party of the state governor since 1961 (Klarner 2013). This is dummy coded as one for Democratic and zero otherwise. These data also include Right to Work laws since 1911 (Boehmke and Skinner 2012). These laws allow employees of unionized firms covered by collective agreements to refuse paying union dues, thereby limiting resources for unions to mobilize or organize workers (Macdonald 2019). This is dummy coded as one for having a Right to Work law and zero otherwise.

Individual and state characteristics are intended to be as broad as possible to test whether union effects extend to the whole working community. Consequently, estimates using the GSS and CPS are from all adult (18 and older) employed workers.

## Models

Using these data, I estimate separate regressions on each indicator of SFRI, *R*, for respondent *i* (*i* = 1*,...,N*) for a given state, *s*, and year, *t*. The key predictor is union density, *U*, for each respondent in the given state and year. Individual covariates for age, sex, race, marital status, education, and employment status are included in the vector *C*. State covariates for state population and the proportion of workers that are young, female, non-white, not currently married, with 12 or fewer years of education, and self-employment are included in vector *K*. Additional covariates including individual union membership, log wage variance, governor party, and Right to Work laws are included in vector *E*. I uniquely identify these covariates in vector *E* because they also determine union density and thus “confound” union density effects on SFRI in the model. A LPM can be written as

$$\begin{array}{l} R_{ist}=\beta_{1}U_{st}+\beta_{2}C_{ist}+\beta_{3}K_{st}+\beta_{4}E_{ist}\\ \;\;\;\;\;\;\;\;\;\;\;\;+\eta_{s}+\alpha_{t}+\varepsilon_{ist} \end{array}$$

where *η*_*s*_ is a state effect, *α*_*t*_ is a time effect, and *ε*_*ist*_ is an individual random error. I also ran parallel analyses using logit models that produced similar results, yet LPMs are selected because the results are easier to interpret. I experiment with random- and fixed-effects approaches for state and year. Fixed-effects models improve causal inference by controlling for unobserved state characteristics that are stable over time by focusing on changes within states. However, fixed-effects models may hold unrealistic assumptions that past union density does not affect current SFRI, referred to as carryover effects, and past SFRI does not affect current union density, referred to as feedback effects. Effective union organizing often takes years to become established and influence local attitudes (McAlevey 2016). Others argue that historically non-egalitarian regions in the US are resistant to union organizing, suggesting the reverse causal direction that attitudinal shifts “kill” unions (Hogler, Hunt and Weiler 2015).

Marginal structural models (MSMs) relax these assumptions using inverse probability treatment (IPT) weights (VanderWeele 2009; Wodtke 2018; Hernan and Robins 2020). IPT weights are used to construct a pseudo-sample in which the regional proportion of union members at each period is unrelated to the observed past. The IPT weights can be expressed as

$$w_{st}=\prod_{t=0}^{t}\frac{f\left(U_{st}|{\underline{U}}_{st-1},K_{st=0}\right)}{f\left(U_{st}|{\underline{U}}_{st-1},{\underline{R}}_{st-1},{\underline{K}}_{st-1}\right)}$$

where underbars denote covariate histories (i.e., *R*_*st*_ =*R*_*st*=0_, ..., *R*_*st*=*t*_) and thus *f*(*U*_*st*_ | U_*st*_−_1_,R_*st*_−_1_,K_*st*_−_1_) denotes the conditional probability of union membership, *U*_*st*_, given a state’s observed history of the proportion of union members, average SFRI, and state covariates through time *t* −1.

I use IPT weights to regress two over-time estimates of union membership proportion on average agreement and strong agreement that the government (1) should reduce income differences (i.e., increase redistribution) and (2) improve the living conditions of the poor, *R*_*st*_, estimated for each state and year. These questions are selected because they have larger samples and are included in more survey waves than other indicators of SFRI. The first predictor, periodized union membership, $P_{st}^{u}$ , is an estimate of average union membership for each state across time *t*−9 through time *t* (i.e., the current and preceding 9 years). Second, the average change in union membership, $D_{st}^{u}$ , is an estimate of the average decrease (or increase) in union membership for each state over the same moving 10-year period. Vector *K* includes baseline state covariates. MSMs take the log binomial form

$$\begin{array}{l} \log\left(R_{st}\left(P_{st}^{u},D_{st}^{u}\right)\right)=g\left(\beta ,P_{st}^{u},D_{st}^{u}\right)\\ \;\;\;\;\;\;\;\;\;\;\;\;\;\;\;\;\;\;\;\;\;\;\;\;\;\;\;\;\;\;\;\;\;\;\;\;\;\;\;\;\;\;\;\;\;\;+\Theta K_{st=0}+\alpha_{t} \end{array}$$

where β is a vector-valued parameter, *α*_*t*_ is a time effect, g() is a function of 10-years average and average decline (or increase) in union membership. Using the above models, different indicators of state unionization and SFRI are individually compared.

## Findings

Table 1 displays average characteristics for 51 states (including DC) equally divided by low, medium, and high union density between 1973 and 2016. The lowest 17 states have an average of five to 10% union membership for this period, “medium” includes 10–18%, and “high” includes 18–28%. Proportions for young, female, non-white, non-married, and self-employed workers show that states have similar demographics across union density. However, high union density states have notably fewer workers with postsecondary education, lower wage inequality, more democratic governors, and fewer instances of Right to Work laws compared to states with medium and low union density. Such variation hints at a potential dampening influence on union density effects because they are also hypothesized to determine SFRI.

**Table 1.** Descriptive Statistics (Averages) by States With Low, Medium, and High Union Density.

Variable	Low Union Density	Medium Union Density	High Union Density	Sample
Union membership	0.07	0.14	0.24	
Union locals per 500,000	23.52	45.81	47.34	
Young	0.26	0.26	0.30	
Female	0.46	0.46	0.44	
Non-white	0.16	0.13	0.15	
Non-married	0.39	0.38	0.35	
Non-postsecondary	0.43	0.47	0.54	
Self-employed	0.12	0.12	0.10	
Log wage variance	0.34	0.33	0.31	
Democratic Governor	0.38	0.59	0.58	
Right to work	0.79	0.30	0.18	
Income gap too large	0.59	0.64	0.64	3,395
Inequality benefits rich	0.50	0.55	0.57	3,284
Inequality not necessary	0.50	0.54	0.49	3,332
More redistribution	0.44	0.47	0.46	25,010
More help for poor	0.26	0.27	0.31	19,562
More taxes for rich	0.44	0.52	0.50	4,156
Less taxes for poor	0.63	0.62	0.68	3,562

The final seven rows of Table 1 show average SFRI and sample sizes. The farthest right column shows that support for more redistribution and help for the poor have the largest samples and thus allow for more precise estimations. Workers in low compared to medium union density states are four to five percentage points less likely to agree that the income gap is too large, inequality benefits the rich, and inequality is not necessary for prosperity. Fewer workers in low compared to high union density states support more progressive taxation, increasing redistribution, and helping the poor. In sum, states with low union density tend to exhibit the lowest SFRI.

**Figure 1.**
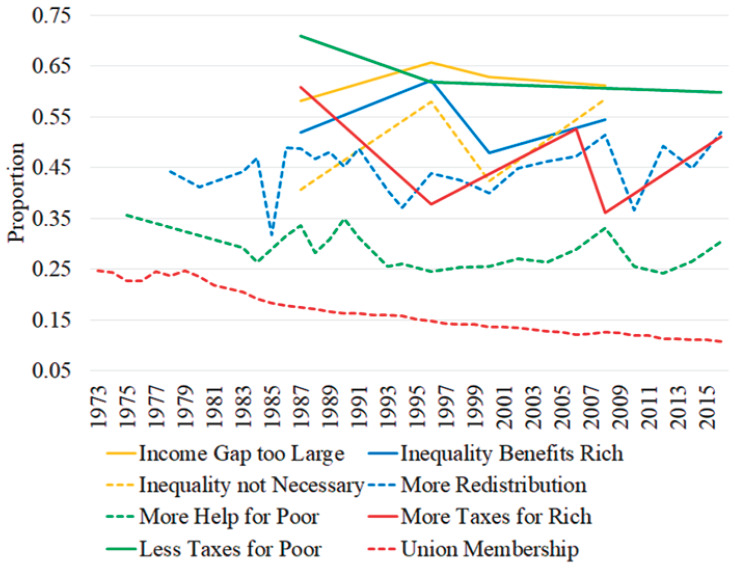
Union membership and SFRI over time.

The trend lines in Figure 1 show annual national union membership and average agreement (including strong agreement) with each indicator of SFRI. The trend line for union membership shows a gradual decline from about 25 to 10% of workers. However, both support for redistribution and helping the poor increased during late 1980s and again after the 2000s despite declining unionization. Other indicators of SFRI (beginning in 1987) show similar peaks and valleys in average support, inconsistent with union decline. While these estimates mask differences between individuals and states, they do not show the gradual drop in SFRI over time that some perspectives on unionization may assume.

**Figure 2.**
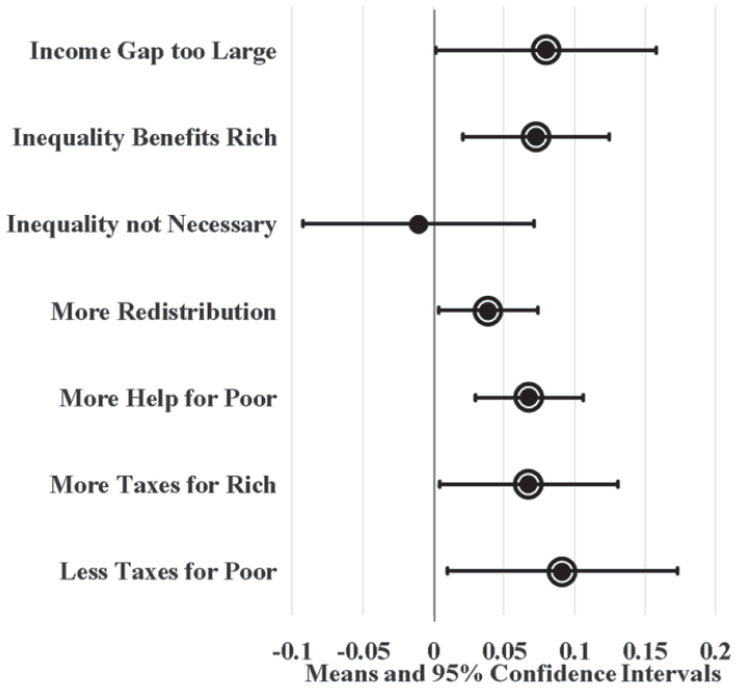
Unadjusted state union membership effects on SFRI. Note: Circle borders denote statistical significance at the 0.05 level.

Figure 2 displays LPM results for state union membership in a dot and whisker graph. Dots indicate the average effect of a 23 percentage point increase in union membership and whiskers show 95% confidence intervals. The value 23 percentage points measures a realistically large change, representing the gap between the fifth largest state union membership (in New Jersey) and lowest state union membership (in South Carolina) averaged across all years during this period. This value also represents the fifth largest decline in union membership among states during this period (which was experienced by Pennsylvania). The circle borders around the dots denote statistical significance at the 0.05 level. Assuming union membership increases SFRI, dots should fall to the right of the zero, with circle borders and small whiskers indicating that the predictions are precise; this is largely what results in Figure 2 show. For example, states with a high proportion of union members, like New Jersey, are estimated to have four and seven percentage points more support for redistribution and helping the poor, respectively, compared to states with a low proportion of union members, like South Carolina. Models also estimate that high union membership states have workers that are seven to nine percentage points more likely to agree that the income gap is too large, inequality benefits the rich, and taxes should be increased for the rich and reduced for the poor compared to low union membership states.

**Figure 3.**
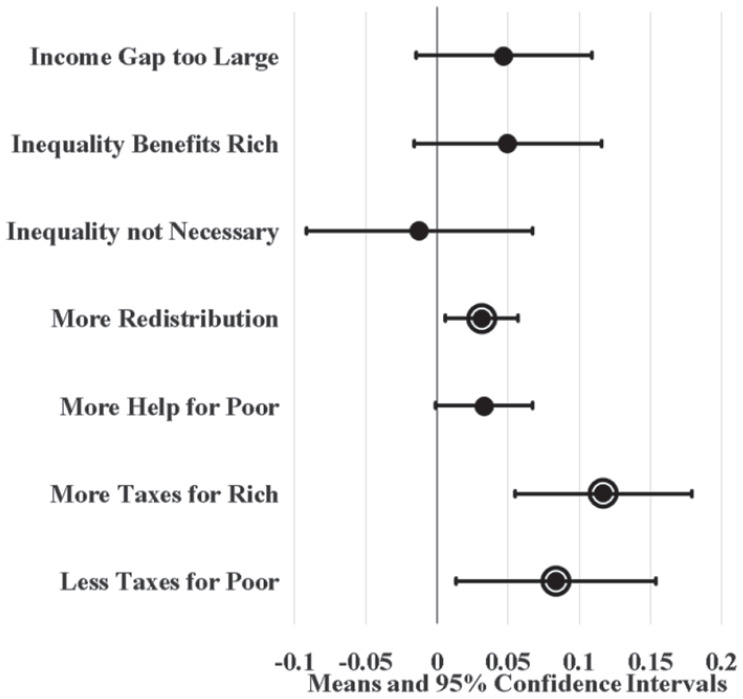
Unadjusted state union local effects on SFRI. Note: Circle borders denote statistical significance at the 0.05 level.

Figure 3 displays the unadjusted attitudinal effects of increasing union locals per 500,000 population by 52. The value 52 measures a realistically large change, representing a considerable difference between high and low union density states and the fifth largest decline in union locals. Union locals represent an alternative measure of union density. Linear probability models suggest that states with a high density of union locals have workers that are three percentage points more likely so support redistribution and eight to 12 percentage points more likely to support tax progressivity compared to states with low union local density. Comparing Figures 3 and 2 suggests that union local effects on SFRI tend to be less precise than union membership effects. Yet, results from both unadjusted LMPs suggest that states with high union density have higher (about three to 12 percentage points) SFRI compared to low union density states.

**Figure 4.**
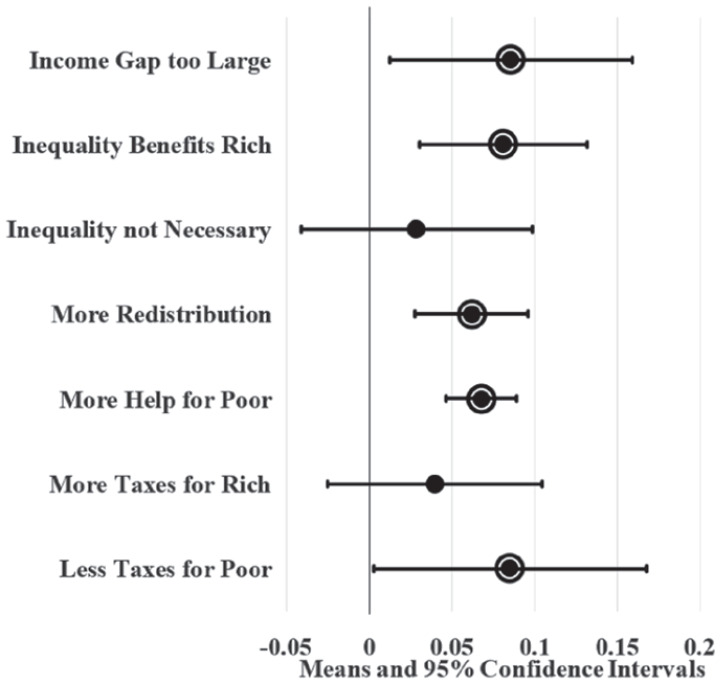
Adjusted state union membership effects on SFRI. Note: Circle borders denote statistical significance at the 0.05 level.

**Figure 5.**
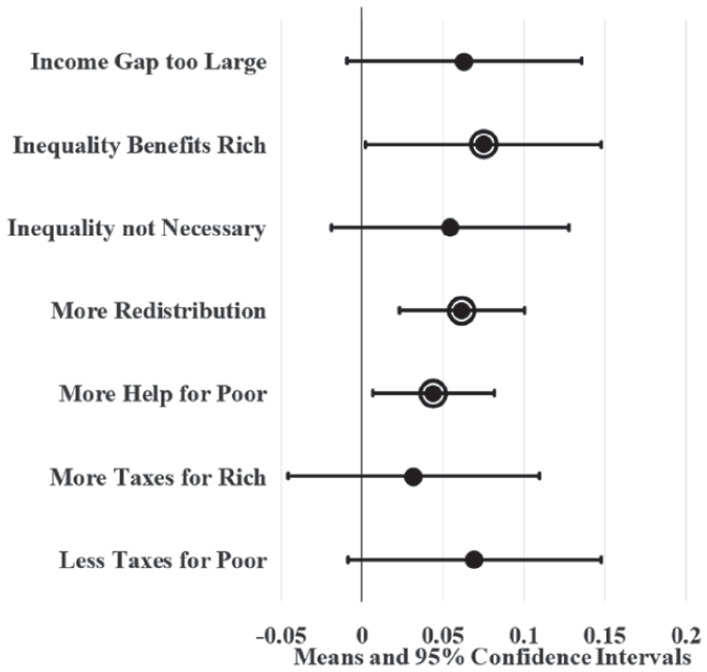
Adjusted state union local effects on SFRI. Note: Circle borders denote statistical significance at the 0.05 level.

Figures 4 and 5 display LPM estimates of union density effects while controlling for individual (age, sex, race, marital status, education, and employment status) and state (population and the proportion of workers that are young, female, non-white, not currently married, with 12 or fewer years of education, and self-employment) covariates. Given that Washington, DC is politically unique and may be an outlier, I ran parallel LPMs that variously leave out and control for DC using a dummy variable; union density estimates from these parallel models did not significantly differ from results displayed here (available upon request). I also ran parallel LPMs with additional covariates (individual union membership, log wage variance, party governor, and Right to Work laws) that predict similar yet less precise union density effects (available in online supplement). Since these additional covariates complicate (and possibly confound or mediate) union density effects in the model, Figures 4 and 5 display the effects of covariate-adjusted LPMs without them.

Figure 4 shows that high union membership states, like New Jersey, have workers that are six to nine percentage points more likely to agree that inequality is too large, inequality benefits the rich, redistribution should be increased, there should be more help for the poor, and the poor should be taxed less compared to low union membership states, like South Carolina, after controlling for covariates. Likewise, Figure 5 shows that states with a high density of union locals have workers that are four to seven percentage points more likely to agree that inequality benefits the rich, the government should increase redistribution, and there should be more help for the poor compared to states with a low density of union locals.

Comparing union density to demographic and other compositional effects on SFRI provides some context for interpreting union density effect sizes. For example, parallel adjusted models show that non-white workers are 14 percentage points more likely to support redistribution and 16 percentage points more likely to support helping the poor compared to white workers, net of union density and individual and regional covariates. Female workers are five percentage points more likely to support redistribution and four percentage points more likely to support helping the poor compared to male workers. The fifth largest within-state change in the proportion of non-white workers is 20 percentage points during this period. A 20 percentage point increase in non-white workers is associated with a one percentage point decrease in support for redistribution, net of covariates. None of the adjusted racial composition effects on SFRI are statistically significant at the 0.05 level. The fifth largest change in state log wage variance during this period is 0.4. Parallel models estimate that a large increase in wage variance is associated with a six percentage point increase in support for helping the poor and seven percentage point increase in redistribution support, net of covariates and union density. Effect sizes of union density on SFRI tend to fall between parallel effect sizes for race and gender and are comparable to inequality effects hypothesized by the median-voter model, suggesting an overall moderate effect.

**Figure 6.**
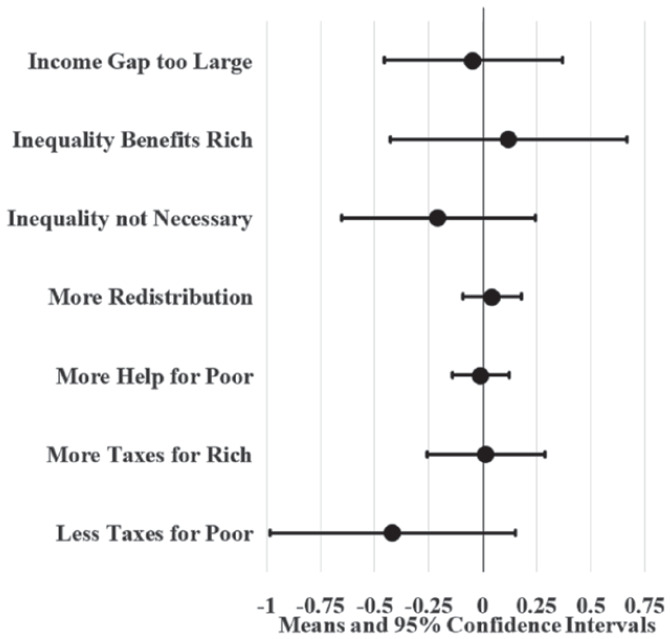
Fixed state union membership effects on SFRI

**Figure 7.**
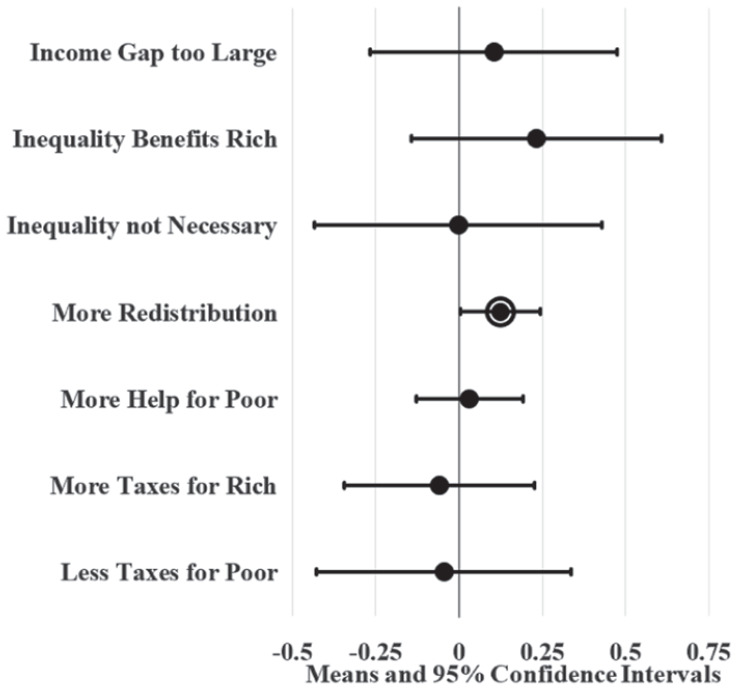
Fixed state union local effects on SFRI. Note: Circle borders denote statistical significance at the 0.05 level.

I also estimate LPMs that include individual and state covariates and fixed-effects for state and survey year, visualized in Figures 6 and 7. Fixed-effects test whether union density remains associated with SFRI after accounting for unobserved characteristics of states, thereby isolating effects of changes in union density within states and suggesting a causal relationship between the two. These models help account for contextual effects, such as deindustrialization, shift towards business unionism, and other changes within states during this period. Yet, few circle borders and large whiskers for most dots in Figures 6 and 7 show that the fixed-effects models do not produce precise estimates of union decline effects on SFRI. The estimates displayed in Figure 7 suggest that had union density not declined in Pennsylvania, for example, and instead remained stable, Pennsylvanian workers would have 12 percentage point higher support for redistribution in 2016. However, the standard errors for this estimate are four times larger than estimates for the same attitude in the unadjusted and adjusted models. I also run parallel models that include linear (instead of fixed) year effects and additional (vector *E*) covariates that provide similar results (available upon request). These results may cast doubt on assumptions that union density directly causes SFRI, yet there may be too little variation in unions or SFRI within states to reliably predict union density effects with fixed-effects models.

While fixed-effects improve causal inference by accounting for unobserved time-invariant confounders, they assume past treatments do not directly influence current outcome, and past outcomes do not directly affect current treatment (Imai and Kim 2019). This assumption may not be representative of dynamic relationships, such as in cases of plausible reverse causality. MSMs, on the other hand, relax assumptions related to carryover and feedback effects (such as levels of support for redistribution and union density at earlier time points) yet do not account for time-invariant confounders. Evaluating which assumptions are less credible is complex and no current method accounts for both unobserved time-invariant confounders and past treatments without making additional assumptions (Imai and Kim 2019). Rather than emphasizing one approach, I estimate MSMs to complement the results from fixed-effects and other models.

**Figure 8.**
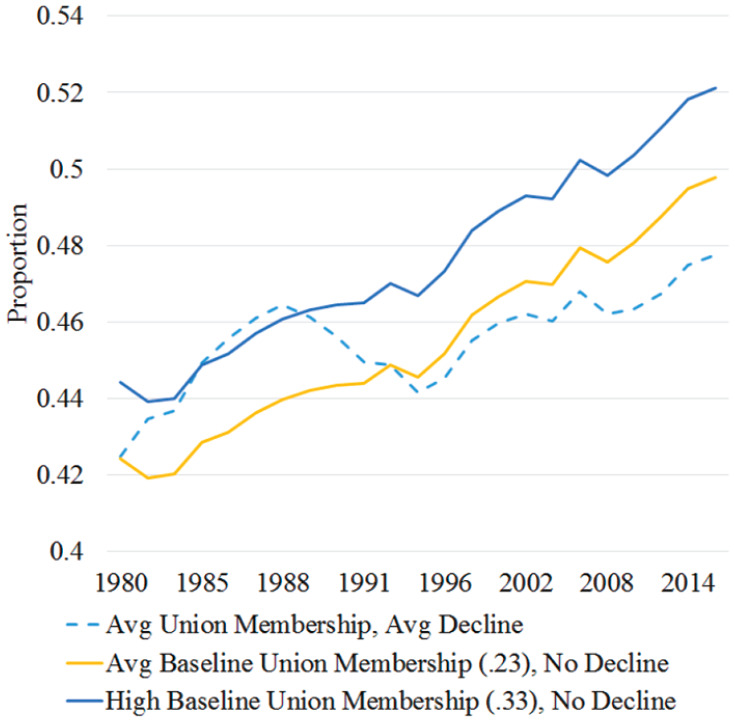
Estimated support for redistribution by union membership simulations.

Using MSMs, I estimate union membership effects on support for redistribution over time by balancing time-varying confounders, such as earlier union membership and support for redistribution across treatment levels at each time point. Using these models, Figure 8 displays trend lines that counterfactually estimate what state-wide support for redistribution would have been given different 10-year averages of union membership and decline. The dotted line shows the predicted probability of supporting redistribution in a state with average union trends. The solid lines estimate what predicted probability of supporting this attitude would have been if union membership was average (23%) or high (33%) in 1973 (censored in the graph) and did not change during this period. Models estimate that states with high compared to average baseline union membership without decline have more support for more redistribution by two percentage points, consistent with the unadjusted and covariate-adjusted LPMs. The dotted line shows that estimated support for redistribution increased in states with average union trends by five percentage points between 1980 and 2016, despite notable decline in the 1990s. However, if union membership had not declined, models counterfactually estimate that support for redistribution would have gradually increased by about seven percentage points during this period.

**Figure 9.**
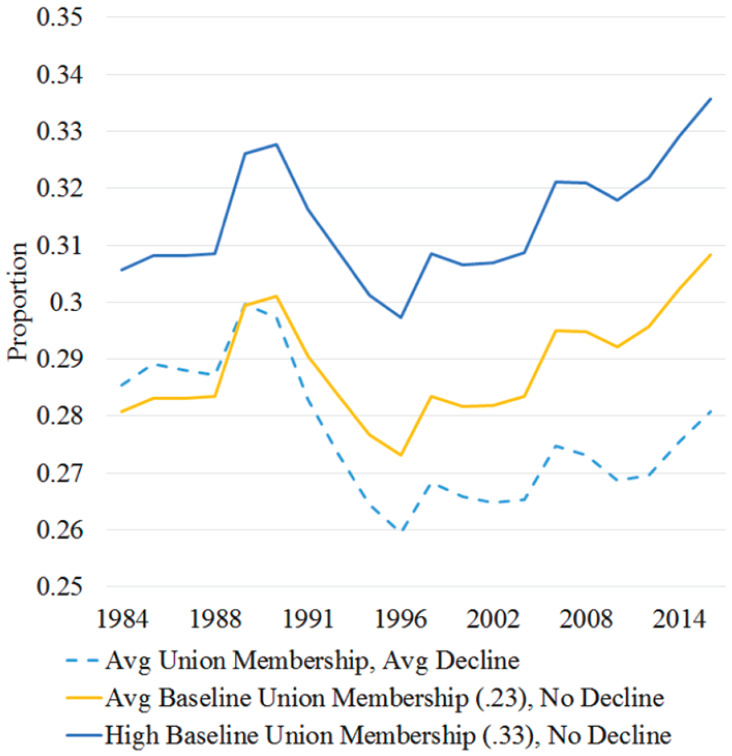
Estimated support for helping the poor by union membership simulations.

Figure 9 likewise visualizes the annual predicted and counterfactual probability of supporting improving the living condition of the poor from MSMs. The gap between yellow and blue trend lines suggests that states with 10% higher union membership (and without decline) have more support for helping the poor by about three percentage points, consistent with the covariate adjusted model. The dotted line shows that estimated support for helping the poor in states with average union membership trends is similar in 1984 and 2016 despite decreasing in the 1990s. However, models counterfactually estimate that if union membership had not declined, support for helping the poor would have increased by about three percentage points over this period.

## Conclusion

Academics, among others, often assume that unions influence inequality attitudes. This belief draws on popular narratives of unions fighting for cultural solidarity among workers and fostering pay norms that hold employers and politicians accountable. This paper evaluates and lends some empirical support for this hypothesis by drawing on representative data from several large surveys, including the GSS, CPS, and CBP data between 1973 and 2016. Results from linear probability, fixed-effects, and MSMs estimate that a large increase in union density moderately increases SFRI by three to 12 percentage points. This addresses an important gap in moral economy and Power Resource research that often uses culture and norms to help explain union density effects on inequality and welfare policy. They suggest that unions foster norms related to compensation that is “too much” or “too little” that, in turn, make employers and politicians fearful of inciting public outcry, thereby reducing income inequality. Until now, scholars have had only limited and conflicting evidence to evaluate whether unions influence markets and policy through culture and norms, or whether other mechanisms such as bargaining and membership on regulatory boards should be emphasized. While this paper does not evaluate such alternative mechanisms, findings suggest that future studies seeking to explain union density effects should seriously consider the mediating role of inequality attitudes proposed by moral economy and Power Resource scholars.

Findings also contribute to research on inequality attitudes. McCall (2013) proposes that narratives that frame income inequality as harmful for social mobility or equitable economic growth increase SFRI. Unions have historically played an important role in such narratives, arguing for the benefits of egalitarianism in protests, newsletters, and Labor Day parade speeches, among other platforms (Dray 2011; Western and Rosenfeld 2011). Findings lend support for this approach by suggest that institutions involved in inequality narratives, such as unions, are important for explaining SFRI. Union density effects on SFRI are comparable to factors emphasized by the median-voter and thermostatic model, such as income inequality, race, and other demographic characteristics. Findings also point to important nuances in the relationship between union density and SFRI. Union density has the largest positive effects on attitudes that the income gap is too large, inequality exists to benefit the rich, and that taxes should be reduced on the poor after introducing covariates. These attitudes may be considered less controversial as “benefiting the rich” does not necessarily imply negative connotations towards inequality and reducing taxes on the poor is widely popular and consistent with both Republican campaigns for fewer taxes and Democratic campaigns to alleviant stress on the poor. Questions that implicate the rich or inequality as harmful, such as increasing taxes on the rich and whether inequality is necessary for prosperity are less frequently associated with union density in the models. While unions appear to increase some indicators of SFRI, results suggest that they may be less effective at introducing inequality narratives that frame the rich as “undeserving.”

Findings in this analysis may be influenced by the unique context of union decline in the US. While some unions continue to practice whole worker and community organizing, many have been restricted to within-firm bargaining strategies since the 1980s. Fewer union members, regulations on union mobilization, fewer manufacturing industries, and Right to Work laws restrict unions’ capacity to influence attitudes among a wide variety of workers. (Black 2005; Mollona 2009; Rosenfeld 2014). Yet, research shows that unions continue to reduce state-level poverty among unionized and nonunionized households despite their decline, suggesting that unions may continue to influence norms or engage in other activity that extends their reach beyond unionized firms (Brady, Baker, and Finnigan 2013). It is also possible that union density may have stronger effects on SFRI in countries like Belgium, Finland, and other Ghent style governments where organized labor has experienced less decline, welfare policies are in part administered through union membership, and bargaining is more centralized within politics. However, others argue union density effects on SFRI may be weaker in Ghent counties because many join unions to access employment insurance instead of ideological factors (Mosimann and Pontusson 2017). Thus, moderate union density effects on SFRI estimated in this analysis may be unique to the US and vary by period and country.

Findings provide resources for future research on union density and SFRI within smaller aggregates. Conducting analyses at the state level allows scholars to draw across a variety of surveys, from the GSS, CPS, and others and subsequently control for a wide range of covariates. Yet, union organizing may be more salient in countries and metropolitan statistical areas where residents may directly observe strikes, protests, campaign posters, and other types of union intervention. While union local data are less widely used as a measure of “union density,” my findings show that their effects on SFRI are largely consistent with and provide a reliable alternative to the more conventional union membership effects. How union locals influence inequality attitudes and related characteristics in smaller regions remains a significant gap in the literature.

This research also answers a call from scholars to pay more attention to “how intersubjectively shared meaning structures (e.g., scripts, narratives, repertoires, and symbolic boundaries) come to enable and constrain behaviours” within explanations of income inequality (Lamont, Beljean and Clair 2014). Competing perspectives on income inequality tend to focus on coinciding market characteristics, such as the changing demographics of workers, economic restructuring (e.g., the rise of temporary work), weakening political protections for workers rights (e.g., stagnation of the minimum wage), and the growth of capital through globalization (Morris and Western 1999). The moral economy perspective and Power Resource Theory are unique because they identify union-related culture (i.e., egalitarian attitudes) as a central process limiting the growth of wage dispersion. By assessing the assumed relationship between union density and SFRI, my study provides an important step in developing an empirical basis for understanding the cultural mechanisms of rising inequality.

## Supplemental Material

sj-pdf-1-scu-10.1177_23294965221089914 – Supplemental material for State Union Density Effects on Workers’ Support for Reducing Income Inequality, 1973-2016 Click here for additional data file. Supplemental material, sj-pdf-1-scu-10.1177_23294965221089914 for State Union Density Effects on Workers’ Support for Reducing Income Inequality, 1973-2016 by Shawn Perron in Social Currents
