# Supplementary material for: State Union Density Effects on Workers’ Support for Reducing Income Inequality, 1973-2016
Source: Soc Curr. 2022 May 26;9(4):369–86. doi: 10.1177/23294965221089914 (PMC9279886; doi:10.1177/23294965221089914)
Supplement: sj-pdf-1-scu-10.1177_23294965221089914 – Supplemental material for State Union Density Effects on Workers’ Support for Reducing Income Inequality, 1973-2016 [file sj-pdf-1-scu-10.1177_23294965221089914.pdf]

APPENDIX (ONLINE SUPPLEMENT)

APPENDIX A: UNION MEMBERSHIP EFFECTS ON SFRI

Table A1. LPM Estimates of Union Membership Effects on Agreement That Income Differences Too Large

	Model 1 Unadjusted	*Model 2a Adjusted*	*Model 2b Adjusted*[1]	*Model 3 Fixed-Effects*
	Coefficient (Standard Error)	Coefficient (Standard Error)	Coefficient (Standard Error)	Coefficient (Standard Error)
State Union Membership	0.348*	0.372*	0.332	-0.672
	(0.17)	(0.16)	(0.23)	(0.75)
State (18+) Population		<-0.001	<-0.001	<0.001
		(<0.01)	(<0.01)	(<0.01)
Age		0.002*	0.002*	0.002*
		(<0.01)	(<0.01)	(<0.01)
Female		0.044	0.060*	0.039
		(0.02)	(0.03)	(0.03)
Non-White		0.066*	0.060*	0.077**
		(0.03)	(0.03)	(0.03)
Non-Married		0.056**	0.073**	0.055**
		(0.02)	(0.02)	(0.02)
High School Diploma (Compared to <HS Diploma)		0.008	-0.009	0.015
		(0.03)	(0.03)	(0.03)
Some College		-0.016	-0.023	-0.009
		(0.03)	(0.04)	(0.04)
Post-Secondary Degree		-0.040	-0.058	-0.035
		(0.03)	(0.03)	(0.03)
Non-Self-Employed (Employee)		0.066*	0.068*	0.066*
		(0.03)	(0.03)	(0.03)
Proportion of Young Workers		-0.077	0.082	-0.283
		(0.39)	(0.39)	(0.90)
Proportion of Female Workers		1.049	0.826	0.848
		(1.44)	(1.52)	(2.05)
Proportion of Non-White		-0.212	-0.277*	-0.199

Workers		(0.14)	(0.14)	(0.84)
Proportion of Non-Married Workers		0.392	0.523	0.942
		(0.44)	(0.46)	(0.95)
Proportion of Non-Postsecondary Workers		-0.091	-0.154	0.022
		(0.23)	(0.27)	(0.68)
Proportion of Non-Self Employed-Workers		0.577	0.324	0.314
		(0.48)	(0.52)	(1.20)
Union Member			-0.005	
			(0.03)	
Democratic Governor			0.024	
			(0.02)	
Right to Work Legislation			0.013	
			(0.03)	
Log Wage Variance			-0.177	
			(0.17)	
Year Fixed-Effects?	No	No	No	Yes
State Fixed-Effects?	No	No	No	Yes

1, Additionally adjusted for individual union membership, state governor, Right to Work laws, and log wage variance
**, p<0.05; **, p<0.005; ***, p<0.001*

Table A2. LPM Estimates of Union Membership Effects on Agreement That Inequality Exists to Benefit Rich

	Model 1 Unadjusted	*Model 2a Adjusted*	*Model 2b Adjusted*[1]	*Model 3 Fixed-Effects*
	Coefficient (Standard Error)	Coefficient (Standard Error)	Coefficient (Standard Error)	Coefficient (Standard Error)
State Union Membership	0.316**	0.352**	0.465*	0.048
	(0.11)	(0.11)	(0.19)	(0.98)
State (18+) Population		<-0.001	<-0.001	<-0.001
		(<0.01)	(<0.01)	(<0.01)
Age		<0.001	<0.001	0.001
		(<0.01)	(<0.01)	(<0.01)
Female		-0.018	-0.015	-0.017
		(0.02)	(0.02)	(0.02)
Non-White		0.154***	0.149***	0.153***

		(0.03)	(0.03)	(0.03)
Non-Married		0.036*	0.026	0.034
		(0.02)	(0.02)	(0.02)
High School Diploma (Compared to <HS Diploma)		0.014	0.022	0.022
		(0.03)	(0.04)	(0.03)
Some College		-0.019	-0.002	-0.010
		(0.03)	(0.03)	(0.03)
Post-Secondary Degree		-0.050	-0.047	-0.045
		(0.03)	(0.03)	(0.03)
Non-Self-Employed (Employee)		0.033	0.036	0.028
		(0.02)	(0.03)	(0.03)
Proportion of Young Workers		-0.192	0.086	0.517
		(0.39)	(0.48)	(1.02)
Proportion of Female Workers		0.001	0.875	0.664
		(1.04)	(1.20)	(1.64)
Proportion of Non-White Workers		-0.160	-0.288*	1.810*
		(0.14)	(0.14)	(0.72)
Proportion of Non-Married Workers		0.230	0.099	-0.596
		(0.41)	(0.49)	(1.03)
Proportion of Non-Postsecondary Workers		-0.089	-0.141	-0.078
		(0.24)	(0.26)	(0.57)
Proportion of Non-Self Employed-Workers		0.571	0.125	-0.393
		(0.51)	(0.56)	(0.89)
Union Member			0.048	
			(0.03)	
Democratic Governor			0.015	
			(0.02)	
Right to Work Legislation			0.062	
			(0.03)	
Log Wage Variance			0.248	
			(0.28)	
Year Fixed-Effects?	No	No	No	Yes
State Fixed-Effects?	No	No	No	Yes

1, Additionally adjusted for individual union membership, state governor, Right to Work laws, and log wage variance
**, p<0.05; **, p<0.005; ***, p<0.001*

Table A3. LPM Estimates of Union Membership Effects on Agreement That Inequality Not Necessary for Prosperity

	Model 1 Unadjusted	*Model 2a Adjusted*	*Model 2b Adjusted*[1]	*Model 3 Fixed-Effects*
	Coefficient (Standard Error)	Coefficient (Standard Error)	Coefficient (Standard Error)	Coefficient (Standard Error)
State Union Membership	-0.046	0.125	-0.035	-0.908
	(0.18)	(0.15)	(0.21)	(0.94)
State (18+) Population		<-0.001	<-0.001	<-0.001
		(<0.01)	(<0.01)	(<0.01)
Age		0.002*	0.002*	0.002*
		(<0.01)	(<0.01)	(<0.01)
Female		-0.009	-0.010	-0.008
		(0.02)	(0.02)	(0.02)
Non-White		-0.036	-0.010	-0.039
		(0.02)	(0.04)	(0.02)
Non-Married		-0.003	-0.001	-0.006
		(0.02)	(0.02)	(0.02)
High School Diploma (Compared to <HS Diploma)		0.082**	0.092**	0.087***
		(0.02)	(0.03)	(0.02)
Some College		0.173***	0.182***	0.188***
		(0.03)	(0.04)	(0.03)
Post-Secondary Degree		0.207***	0.222***	0.214***
		(0.03)	(0.04)	(0.03)
Non-Self-Employed (Employee)		-0.016	-0.024	-0.011
		(0.03)	(0.03)	(0.03)
Proportion of Young Workers		-0.987*	-0.995*	-0.451
		(0.44)	(0.44)	(1.36)
Proportion of Female Workers		-0.016	0.803	2.236
		(1.19)	(1.26)	(2.11)
Proportion of Non-		0.182	0.121	-1.109

White Workers		(0.13)	(0.15)	(1.09)
Proportion of Non-Married Workers		-0.361	-0.550	-1.537
		(0.55)	(0.58)	(1.33)
Proportion of Non-Postsecondary Workers		-0.417	-0.380	-0.127
		(0.28)	(0.28)	(1.03)
Proportion of Non-Self Employed-Workers		0.236	0.128	-0.509
		(0.53)	(0.54)	(1.69)
Union Member			0.001	
			(0.03)	
Democratic Governor			0.045	
			(0.02)	
Right to Work Legislation			-0.027	
			(0.03)	
Log Wage Variance			0.171	
			(0.27)	
Year Fixed-Effects?	No	No	No	Yes
State Fixed-Effects?	No	No	No	Yes

1, Additionally adjusted for individual union membership, state governor, Right to Work laws, and log wage variance
**, p<0.05; **, p<0.005; ***, p<0.001*

Table A4. LPM Estimates of Union Membership Effects on Agreement That Government Should Reduce Income Differences (Support for Income Redistribution)

	Model 1 Unadjusted	*Model 2a Adjusted*	*Model 2b Adjusted*[1]	*Model 3 Fixed-Effects*
	Coefficient (Standard Error)	Coefficient (Standard Error)	Coefficient (Standard Error)	Coefficient (Standard Error)
State Union Membership	0.169*	0.270***	0.253*	0.156
	(0.08)	(0.07)	(0.11)	(0.26)
State (18+) Population		<-0.001	<-0.001	<-0.001
		(<0.01)	(<0.01)	(<0.01)
Age		-0.002***	-0.002***	-0.002***
		(<0.01)	(<0.01)	(<0.01)

Female		0.055***	0.059***	0.054***
		(0.01)	(0.01)	(0.01)
Non-White		0.137***	0.125***	0.135***
		(0.01)	(0.01)	(0.01)
Non-Married		0.044***	0.045***	0.044***
		(0.01)	(0.01)	(0.01)
High School Diploma (Compared to <HS Diploma)		-0.081***	-0.075***	-0.080***
		(0.01)	(0.01)	(0.01)
Some College		-0.122***	-0.116***	-0.117***
		(0.01)	(0.01)	(0.01)
Post-Secondary Degree		-0.165***	-0.157***	-0.164***
		(0.01)	(0.01)	(0.01)
Non-Self-Employed (Employee)		0.081***	0.074***	0.080***
		(0.01)	(0.01)	(0.01)
Proportion of Young Workers		-0.338*	-0.293	-0.056
		(0.13)	(0.15)	(0.41)
Proportion of Female Workers		-0.085	0.411	-0.436
		(0.38)	(0.40)	(0.47)
Proportion of Non-White Workers		-0.066	-0.086	-0.141
		(0.05)	(0.06)	(0.19)
Proportion of Non-Married Workers		0.202	0.143	-0.376
		(0.16)	(0.17)	(0.28)
Proportion of Non-Postsecondary Workers		0.023	0.101	-0.016
		(0.08)	(0.09)	(0.26)
Proportion of Non-Self Employed-Workers		-0.143	0.235	0.012
		(0.17)	(0.18)	(0.46)
Union Member			0.050***	
			(0.01)	
Democratic Governor			0.024*	
			(0.01)	
Right to Work Legislation			0.005	
			(0.02)	
Log Wage Variance			0.185*	
			(0.08)	
Year Fixed-Effects?	No	No	No	Yes

State Fixed-Effects?	No	No	No	Yes

1, Additionally adjusted for individual union membership, state governor, Right to Work laws, and log wage variance
**, p<0.05; **, p<0.005; ***, p<0.001*

Table A5. LPM Estimates of Union Membership Effects on Agreement That Government Should Improve Living Standard for the Poor

	Model 1 Unadjusted	*Model 2a Adjusted*	*Model 2b Adjusted*[1]	*Model 3 Fixed-Effects*
	Coefficient (Standard Error)	Coefficient (Standard Error)	Coefficient (Standard Error)	Coefficient (Standard Error)
State Union Membership	0.294***	0.295***	0.271**	-0.001
	(0.08)	(0.05)	(0.09)	(0.30)
State (18+) Population		<-0.001	<-0.001	<0.001
		(<0.01)	(<0.01)	(<0.01)
Age		-0.002***	-0.002***	-0.002***
		(<0.01)	(<0.01)	(<0.01)
Female		0.041***	0.040***	0.042***
		(0.01)	(0.01)	(0.01)
Non-White		0.156***	0.150***	0.154***
		(0.01)	(0.01)	(0.01)
Non-Married		0.052***	0.052***	0.052***
		(0.01)	(0.01)	(0.01)
High School Diploma (Compared to <HS Diploma)		-0.084***	-0.085***	-0.082***
		(0.01)	(0.01)	(0.01)
Some College		-0.104***	-0.105***	-0.101***
		(0.02)	(0.01)	(0.02)
Post-Secondary Degree		-0.107***	-0.108***	-0.109***
		(0.01)	(0.01)	(0.01)
Non-Self-Employed (Employee)		0.032***	0.032***	0.032***
		(0.01)	(0.01)	(0.01)
Proportion of Young Workers		-0.078	-0.034	0.416
		(0.15)	(0.14)	(0.37)
Proportion of Female		-0.450	-0.362	-0.094

Workers				
		(0.35)	(0.35)	(0.56)
Proportion of Non-White Workers		0.008	-0.008	0.078
		(0.06)	(0.06)	(0.25)
Proportion of Non-Married Workers		0.573**	0.588**	0.389
		(0.18)	(0.18)	(0.30)
Proportion of Non-Postsecondary Workers		0.288***	0.344***	0.258
		(0.08)	(0.10)	(0.23)
Proportion of Non-Self Employed-Workers		0.093	0.167	0.588
		(0.21)	(0.23)	(0.35)
Union Member			0.031*	
			(0.01)	
Democratic Governor			0.006	
			(0.01)	
Right to Work Legislation			<0.001	
			(0.02)	
Log Wage Variance			0.156*	
			(0.07)	
Year Fixed-Effects?	No	No	No	Yes
State Fixed-Effects?	No	No	No	Yes

1, Additionally adjusted for individual union membership, state governor, Right to Work laws, and log wage variance
**, p<0.05; **, p<0.005; ***, p<0.001*

Table A6. LPM Estimates of Union Membership Effects Agreement That Taxes Are Much Too Low for the Rich

	Model 1 Unadjusted	*Model 2a Adjusted*	*Model 2b Adjusted*[1]	*Model 3 Fixed-Effects*
	Coefficient (Standard Error)	Coefficient (Standard Error)	Coefficient (Standard Error)	Coefficient (Standard Error)
State Union Membership	0.293*	0.173	0.007	-0.378
	(0.14)	(0.14)	(0.23)	(0.52)
State (18+) Population		<-0.001**	<-0.001*	<-0.001

		(<0.01)	(<0.01)	(<0.01)
Age		0.004***	0.004***	0.003***
		(<0.01)	(<0.01)	(<0.01)
Female		0.027	0.023	0.024
		(0.02)	(0.02)	(0.02)
Non-White		-0.059**	-0.060**	-0.062***
		(0.02)	(0.02)	(0.02)
Non-Married		0.034*	0.024	0.032
		(0.02)	(0.02)	(0.02)
High School Diploma (Compared to <HS Diploma)		0.087***	0.107***	0.089***
		(0.02)	(0.02)	(0.02)
Some College		0.115***	0.127***	0.127***
		(0.02)	(0.02)	(0.02)
Post-Secondary Degree		0.064**	0.070**	0.069**
		(0.02)	(0.02)	(0.02)
Non-Self-Employed (Employee)		0.069**	0.047	0.064*
		(0.02)	(0.03)	(0.02)
Proportion of Young Workers		0.575	0.552	0.697
		(0.39)	(0.48)	(0.77)
Proportion of Female Workers		-2.281*	-2.718**	-2.092
		(0.98)	(1.01)	(1.34)
Proportion of Non-White Workers		-0.179	-0.167	0.971
		(0.12)	(0.13)	(0.54)
Proportion of Non-Married Workers		-0.072	-0.209	-0.789
		(0.42)	(0.45)	(0.60)
Proportion of Non-Postsecondary Workers		-0.097	-0.131	-0.128
		(0.16)	(0.25)	(0.40)
Proportion of Non-Self Employed-Workers		-1.396***	-1.404**	-1.545
		(0.38)	(0.50)	(0.87)
Union Member			0.001	
			(0.03)	
Democratic Governor			0.046	
			(0.02)	
Right to Work Legislation			-0.024	
			(0.04)	

Log Wage Variance			-0.183	
			(0.19)	
Year Fixed-Effects?	No	No	No	Yes
State Fixed-Effects?	No	No	No	Yes

1, Additionally adjusted for individual union membership, state governor, Right to Work laws, and log wage variance
**, p<0.05; **, p<0.005; ***, p<0.001*

Table A7. LPM Estimates of Union Membership Effects Agreement That Taxes Are Much Too High for the Poor

	Model 1 Unadjusted	*Model 2a Adjusted*	*Model 2b Adjusted[1]*	*Model 3 Fixed-Effects*
	Coefficient (Standard Error)	Coefficient (Standard Error)	Coefficient (Standard Error)	Coefficient (Standard Error)
State Union Locals per 500,000 Population	0.397*	0.370*	0.421	-1.717*
	(0.18)	(0.18)	(0.31)	(0.79)
State (18+) Population		<-0.001	<-0.001	<0.001
		(<0.01)	(<0.01)	(<0.01)
Age		0.001	0.002*	0.001
		(<0.01)	(<0.01)	(<0.01)
Female		0.028	0.029	0.027
		(0.02)	(0.02)	(0.02)
Non-White		0.083***	0.065*	0.085***
		(0.02)	(0.03)	(0.02)
Non-Married		0.092***	0.096***	0.089***
		(0.02)	(0.02)	(0.02)
High School Diploma (Compared to <HS Diploma)		-0.061**	-0.076*	-0.065**
		(0.02)	(0.03)	(0.02)
Some College		-0.082**	-0.095*	-0.085**
		(0.03)	(0.04)	(0.03)
Post-Secondary Degree		-0.133***	-0.149***	-0.135***
		(0.03)	(0.03)	(0.03)
Non-Self-Employed (Employee)		-0.020	-0.025	-0.021
		(0.02)	(0.03)	(0.02)
Proportion of Young Workers		0.689	0.754	-0.870
		(0.37)	(0.54)	(0.96)
Proportion of Female		0.072	-0.039	-1.908

Workers				
		(1.00)	(1.51)	(1.67)
Proportion of Non-White Workers		0.093	0.256	0.688
		(0.15)	(0.17)	(0.61)
Proportion of Non-Married Workers		-0.461	-0.452	1.184
		(0.35)	(0.36)	(1.01)
Proportion of Non-Postsecondary Workers		-0.193	-0.399	0.056
		(0.16)	(0.22)	(0.61)
Proportion of Non-Self Employed-Workers		-0.943	-1.103	-1.346
		(0.63)	(0.78)	(1.45)
Union Member			0.065*	
			(0.03)	
Democratic Governor			-0.007	
			(0.03)	
Right to Work Legislation			0.002	
			(0.04)	
Log Wage Variance			-0.549	
			(0.31)	
Year Fixed-Effects?	No	No	No	Yes
State Fixed-Effects?	No	No	No	Yes

1, Additionally adjusted for individual union membership, state governor, Right to Work laws, and log wage variance
**, p<0.05; **, p<0.005; ***, p<0.001*

APPENDIX B: UNION LOCAL EFFECTS ON SFRI

Table A8. LPM Estimates of Union Local Effects on Agreement That Income Differences Too Large

	Model 1 Unadjusted	*Model 2a Adjusted*	*Model 2b Adjusted[1]*	*Model 3 Fixed-Effects*
	Coefficient (Standard Error)	Coefficient (Standard Error)	Coefficient (Standard Error)	Coefficient (Standard Error)
State Union Locals per 500,000 Population	0.001	0.001	0.001	0.002
	(<0.01)	(<0.01)	(<0.01)	(<0.01)
State (18+) Population		<0.001	<0.001	<0.001

		(<0.01)	(<0.01)	(<0.01)
Age		0.002*	0.002*	0.002*
		(<0.01)	(<0.01)	(<0.01)
Female		0.044	0.060*	0.039
		(0.02)	(0.03)	(0.03)
Non-White		0.066*	0.061*	0.077**
		(0.03)	(0.03)	(0.02)
Non-Married		0.057**	0.074**	0.055**
		(0.02)	(0.02)	(0.02)
High School Diploma (Compared to <HS Diploma)		0.010	-0.007	0.015
		(0.03)	(0.03)	(0.03)
Some College		-0.015	-0.021	-0.009
		(0.03)	(0.04)	(0.04)
Post-Secondary Degree		-0.039	-0.057	-0.035
		(0.03)	(0.03)	(0.03)
Non-Self-Employed (Employee)		0.065*	0.068*	0.067*
		(0.03)	(0.03)	(0.03)
Proportion of Young Workers		-0.047	0.105	-0.162
		(0.39)	(0.40)	(0.90)
Proportion of Female Workers		1.090	0.814	0.846
		(1.46)	(1.53)	(1.95)
Proportion of Non-White Workers		-0.223	-0.265*	-0.355
		(0.14)	(0.13)	(0.88)
Proportion of Non-Married Workers		0.309	0.477	0.748
		(0.48)	(0.50)	(0.92)
Proportion of Non-Postsecondary Workers		-0.202	-0.185	0.033
		(0.26)	(0.32)	(0.72)
Proportion of Non-Self Employed-Workers		0.426	0.263	0.098
		(0.52)	(0.53)	(1.11)
Union Member			-0.004	
			(0.03)	
Democratic Governor			0.026	
			(0.02)	
Right to Work Legislation			-0.009	
			(0.03)	

Log Wage Variance			-0.167<br>(0.16)	
Year Fixed-Effects?	No	No	No	Yes
State Fixed-Effects?	No	No	No	Yes

1, Additionally adjusted for individual union membership, state governor, Right to Work laws, and log wage variance
**, p<0.05; **, p<0.005; ***, p<0.001*

Table A9. LPM Estimates of Union Local Effects on Agreement That Inequality Exists to Benefit Rich

	Model 1 Unadjusted	*Model 2a Adjusted*	*Model 2b Adjusted*[1]	*Model 3 Fixed-Effects*
	Coefficient (Standard Error)	Coefficient (Standard Error)	Coefficient (Standard Error)	Coefficient (Standard Error)
State Union Locals per 500,000 Population	0.001<br>(<0.01)	0.001*<br>(<0.01)	0.002<br>(<0.01)	0.004<br>(<0.01)
State (18+) Population		<0.001<br>(<0.01)	<0.001<br>(<0.01)	<-0.001<br>(<0.01)
Age		0.001<br>(<0.01)	<0.001<br>(<0.01)	0.001<br>(<0.01)
Female		-0.017<br>(0.02)	-0.016<br>(0.02)	-0.017<br>(0.02)
Non-White		0.155***<br>(0.03)	0.150***<br>(0.03)	0.152***<br>(0.03)
Non-Married		0.037*<br>(0.02)	0.027<br>(0.02)	0.034<br>(0.02)
High School Diploma (Compared to <HS Diploma)		0.014<br>(0.03)	0.023<br>(0.04)	0.022<br>(0.03)
Some College		-0.018<br>(0.03)	<-0.001<br>(0.03)	-0.010<br>(0.03)
Post-Secondary Degree		-0.049<br>(0.03)	-0.046<br>(0.03)	-0.044<br>(0.03)
Non-Self-Employed (Employee)		0.033<br>(0.02)	0.035<br>(0.03)	0.028<br>(0.03)
Proportion of Young Workers		-0.147<br>(0.39)	0.159<br>(0.48)	0.628<br>(1.05)
Proportion of Female		0.007	0.859	0.706

Workers				
		(1.10)	(1.26)	(1.61)
Proportion of Non-White Workers		-0.141	-0.248	1.575
		(0.13)	(0.13)	(0.81)
Proportion of Non-Married Workers		0.141	-0.005	-0.785
		(0.41)	(0.45)	(1.03)
Proportion of Non-Postsecondary Workers		-0.212	-0.263	-0.021
		(0.24)	(0.26)	(0.57)
Proportion of Non-Self Employed-Workers		0.495	0.079	-0.460
		(0.52)	(0.59)	(0.92)
Union Member			0.048	
			(0.03)	
Democratic Governor			0.020	
			(0.02)	
Right to Work Legislation			0.047	
			(0.03)	
Log Wage Variance			0.249	
			(0.29)	
Year Fixed-Effects?	No	No	No	Yes
State Fixed-Effects?	No	No	No	Yes

1, Additionally adjusted for individual union membership, state governor, Right to Work laws, and log wage variance
**, p<0.05; **, p<0.005; ***, p<0.001*

Table A10. LPM Estimates of Union Local Effects on Agreement That Inequality Not Necessary for Prosperity

	Model 1 Unadjusted	*Model 2a Adjusted*	*Model 2b Adjusted*[1]	*Model 3 Fixed-Effects*
	Coefficient (Standard Error)	Coefficient (Standard Error)	Coefficient (Standard Error)	Coefficient (Standard Error)
State Union Locals per 500,000 Population	<-0.001	0.001	0.001	0.001
	(<0.01)	(<0.01)	(<0.01)	(<0.01)
State (18+) Population		<-0.001	<-0.001	<-0.001
		(<0.01)	(<0.01)	(<0.01)
Age		0.002*	0.002*	0.002*
		(<0.01)	(<0.01)	(<0.01)

Female		-0.009	-0.010	-0.008
		(0.02)	(0.02)	(0.02)
Non-White		-0.037	-0.010	-0.040
		(0.02)	(0.04)	(0.02)
Non-Married		-0.002	<0.001	-0.006
		(0.02)	(0.02)	(0.02)
High School Diploma (Compared to <HS Diploma)		0.081**	0.091**	0.087***
		(0.02)	(0.03)	(0.02)
Some College		0.173***	0.180***	0.187***
		(0.03)	(0.04)	(0.03)
Post-Secondary Degree		0.206***	0.222***	0.214***
		(0.03)	(0.04)	(0.03)
Non-Self-Employed (Employee)		-0.016	-0.024	-0.011
		(0.03)	(0.03)	(0.03)
Proportion of Young Workers		-0.950*	-0.949*	-0.339
		(0.43)	(0.44)	(1.40)
Proportion of Female Workers		-0.049	0.843	2.195
		(1.20)	(1.25)	(2.00)
Proportion of Non-White Workers		0.239	0.155	-1.221
		(0.14)	(0.16)	(1.06)
Proportion of Non-Married Workers		-0.440	-0.647	-1.709
		(0.54)	(0.56)	(1.37)
Proportion of Non-Postsecondary Workers		-0.497	-0.512	-0.131
		(0.28)	(0.30)	(1.04)
Proportion of Non-Self Employed-Workers		0.330	0.197	-0.761
		(0.52)	(0.56)	(1.62)
Union Member			-0.002	
			(0.03)	
Democratic Governor			0.046	
			(0.02)	
Right to Work Legislation			0.001	
			(0.03)	
Log Wage Variance			0.150	
			(0.27)	
Year Fixed-Effects?	No	No	No	Yes

State Fixed-Effects?	No	No	No	Yes

1, Additionally adjusted for individual union membership, state governor, Right to Work laws, and log wage variance
**, p<0.05; **, p<0.005; ***, p<0.001*

Table A11. LPM Estimates of Union Local Effects on Agreement That Government Should Reduce Income Differences (Support for Income Redistribution)

	Model 1 Unadjusted	*Model 2a Adjusted*	*Model 2b Adjusted[1]*	*Model 3 Fixed-Effects*
	Coefficient (Standard Error)	Coefficient (Standard Error)	Coefficient (Standard Error)	Coefficient (Standard Error)
State Union Locals per 500,000 Population	0.001*	0.001**	0.001*	0.002*
	(<0.01)	(<0.01)	(<0.01)	(<0.01)
State (18+) Population		<-0.001	<-0.001	<-0.001
		(<0.01)	(<0.01)	(<0.01)
Age		-0.002***	-0.002***	-0.002***
		(<0.01)	(<0.01)	(<0.01)
Female		0.054***	0.059***	0.054***
		(0.01)	(0.01)	(0.01)
Non-White		0.137***	0.125***	0.135***
		(0.01)	(0.01)	(0.01)
Non-Married		0.044***	0.046***	0.044***
		(0.01)	(0.01)	(0.01)
High School Diploma (Compared to <HS Diploma)		-0.082***	-0.076***	-0.080***
		(0.01)	(0.01)	(0.01)
Some College		-0.122***	-0.116***	-0.117***
		(0.01)	(0.01)	(0.01)
Post-Secondary Degree		-0.165***	-0.157***	-0.164***
		(0.01)	(0.01)	(0.01)
Non-Self-Employed (Employee)		0.081***	0.074***	0.081***
		(0.01)	(0.01)	(0.01)
Proportion of Young Workers		-0.295*	-0.260	-0.023
		(0.13)	(0.15)	(0.40)
Proportion of Female Workers		-0.269	0.223	-0.317
		(0.38)	(0.39)	(0.47)
Proportion of Non-		-0.036	-0.044	-0.261

White Workers		(0.06)	(0.06)	(0.19)
Proportion of Non-Married Workers		0.173	0.114	-0.436
		(0.17)	(0.18)	(0.28)
Proportion of Non-Postsecondary Workers		-0.044	0.045	0.015
		(0.08)	(0.10)	(0.26)
Proportion of Non-Self Employed-Workers		-0.160	0.226	0.009
		(0.19)	(0.19)	(0.46)
Union Member			0.052***	
			(0.01)	
Democratic Governor			0.027*	
			(0.01)	
Right to Work Legislation			-0.003	
			(0.01)	
Log Wage Variance			0.178*	
			(0.08)	
Year Fixed-Effects?	No	No	No	Yes
State Fixed-Effects?	No	No	No	Yes

1, Additionally adjusted for individual union membership, state governor, Right to Work laws, and log wage variance
**, p<0.05; **, p<0.005; ***, p<0.001*

Table A12. LPM Estimates of Union Local Effects on Agreement That Government Should Improve Living Standard for the Poor

	Model 1 Unadjusted	*Model 2a Adjusted*	*Model 2b Adjusted*[1]	*Model 3 Fixed-Effects*
	Coefficient (Standard Error)	Coefficient (Standard Error)	Coefficient (Standard Error)	Coefficient (Standard Error)
State Union Locals per 500,000 Population	0.001	0.001*	<0.001	0.001
	(<0.01)	(<0.01)	(<0.01)	(<0.01)
State (18+) Population		<0.001	<-0.001	<0.001
		(<0.01)	(<0.01)	(<0.01)
Age		-0.002***	-0.002***	-0.002***
		(<0.01)	(<0.01)	(<0.01)
Female		0.041***	0.040***	0.042***
		(0.01)	(0.01)	(0.01)
Non-White		0.156***	0.150***	0.154***

		(0.01)	(0.01)	(0.01)
Non-Married		0.053***	0.052***	0.052***
		(0.01)	(0.01)	(0.01)
High School Diploma (Compared to <HS Diploma)		-0.084***	-0.084***	-0.082***
		(0.01)	(0.01)	(0.01)
Some College		-0.103***	-0.104***	-0.101***
		(0.02)	(0.01)	(0.02)
Post-Secondary Degree		-0.107***	-0.107***	-0.109***
		(0.01)	(0.01)	(0.01)
Non-Self-Employed (Employee)		0.032***	0.031***	0.032***
		(0.01)	(0.01)	(0.01)
Proportion of Young Workers		-0.065	-0.057	0.444
		(0.15)	(0.14)	(0.36)
Proportion of Female Workers		-0.682	-0.564	-0.048
		(0.38)	(0.37)	(0.57)
Proportion of Non-White Workers		-0.002	-0.002	0.037
		(0.07)	(0.06)	(0.24)
Proportion of Non-Married Workers		0.600**	0.611**	0.370
		(0.19)	(0.19)	(0.30)
Proportion of Non-Postsecondary Workers		0.258**	0.369***	0.261
		(0.08)	(0.10)	(0.23)
Proportion of Non-Self Employed-Workers		-0.003	0.085	0.593
		(0.24)	(0.26)	(0.36)
Union Member			0.034**	
			(0.01)	
Democratic Governor			0.007	
			(0.01)	
Right to Work Legislation			-0.023	
			(0.01)	
Log Wage Variance			0.149*	
			(0.07)	
Year Fixed-Effects?	No	No	No	Yes
State Fixed-Effects?	No	No	No	Yes

1, Additionally adjusted for individual union membership, state governor, Right to Work laws, and log wage variance
**, p<0.05; **, p<0.005; ***, p<0.001*

Table A13. LPM Estimates of Union Local Effects Agreement That Taxes Are Much Too Low for the Rich

	Model 1 Unadjusted	*Model 2a Adjusted*	*Model 2b Adjusted[1]*	*Model 3 Fixed-Effects*
	Coefficient (Standard Error)	Coefficient (Standard Error)	Coefficient (Standard Error)	Coefficient (Standard Error)
State Union Locals per 500,000 Population	0.002***	0.001	<-0.001	-0.001
	(<0.01)	(<0.01)	(<0.01)	(<0.01)
State (18+) Population		<-0.001*	<-0.001*	<-0.001
		(<0.01)	(<0.01)	(<0.01)
Age		0.004***	0.004***	0.003***
		(<0.01)	(<0.01)	(<0.01)
Female		0.027	0.023	0.024
		(0.02)	(0.02)	(0.02)
Non-White		-0.059**	-0.060**	-0.062***
		(0.02)	(0.02)	(0.02)
Non-Married		0.034*	0.024	0.032
		(0.02)	(0.02)	(0.02)
High School Diploma (Compared to <HS Diploma)		0.087***	0.107***	0.089***
		(0.02)	(0.02)	(0.02)
Some College		0.115***	0.127***	0.126***
		(0.02)	(0.02)	(0.02)
Post-Secondary Degree		0.064**	0.070**	0.069**
		(0.02)	(0.02)	(0.02)
Non-Self-Employed (Employee)		0.069**	0.047	0.064*
		(0.02)	(0.03)	(0.02)
Proportion of Young Workers		0.593	0.552	0.722
		(0.38)	(0.48)	(0.84)
Proportion of Female Workers		-2.288*	-2.718**	-2.124
		(0.94)	(1.00)	(1.40)
Proportion of Non-White Workers		-0.174	-0.167	1.008
		(0.13)	(0.14)	(0.59)
Proportion of Non-		-0.115	-0.210	-0.798

Married				
Workers		(0.42)	(0.45)	(0.67)
Proportion of Non-		-0.144	-0.130	-0.167
Postsecondary Workers		(0.16)	(0.26)	(0.39)
Proportion of Non-Self		-1.425***	-1.406**	-1.550
Employed-Workers		(0.39)	(0.51)	(0.85)
Union Member			0.001	
			(0.03)	
Democratic Governor			0.046*	
			(0.02)	
Right to Work			-0.025	
Legislation				
			(0.03)	
Log Wage Variance			-0.183	
			(0.19)	
Year Fixed-Effects?	No	No	No	Yes
State Fixed-Effects?	No	No	No	Yes

1, Additionally adjusted for individual union membership, state governor, Right to Work laws, and log wage variance
**, p<0.05; **, p<0.005; ***, p<0.001*

Table A14. LPM Estimates of Union Local Effects Agreement That Taxes Are Much Too High for the Poor

	Model 1 Unadjusted	*Model 2a Adjusted*	*Model 2b Adjusted*[1]	*Model 3 Fixed-Effects*
	Coefficient (Standard Error)	Coefficient (Standard Error)	Coefficient (Standard Error)	Coefficient (Standard Error)
State Union Locals per	0.002*	0.001	0.001	0.003
500,000 Population	(<0.01)	(<0.01)	(<0.01)	(<0.01)
State (18+) Population		<-0.001	<-0.001	<-0.001
		(<0.01)	(<0.01)	(<0.01)
Age		0.001	0.002*	0.001
		(<0.01)	(<0.01)	(<0.01)
Female		0.028	0.028	0.027
		(0.02)	(0.02)	(0.02)
Non-White		0.084***	0.065*	0.084**
		(0.02)	(0.03)	(0.02)
Non-Married		0.091***	0.096***	0.089***
		(0.02)	(0.02)	(0.02)

High School Diploma (Compared to <HS Diploma)		-0.062**	-0.076*	-0.063**
		(0.02)	(0.03)	(0.02)
Some College		-0.083**	-0.095*	-0.083**
		(0.03)	(0.04)	(0.03)
Post-Secondary Degree		-0.134***	-0.150***	-0.132***
		(0.03)	(0.03)	(0.03)
Non-Self-Employed (Employee)		-0.020	-0.024	-0.021
		(0.02)	(0.03)	(0.02)
Proportion of Young Workers		0.719	0.770	-0.066
		(0.36)	(0.51)	(0.95)
Proportion of Female Workers		0.012	-0.116	-0.489
		(0.98)	(1.47)	(1.99)
Proportion of Non-White Workers		0.100	0.276	0.171
		(0.14)	(0.17)	(0.70)
Proportion of Non-Married Workers		-0.548	-0.546	0.346
		(0.32)	(0.35)	(1.17)
Proportion of Non-Postsecondary Workers		-0.290	-0.463	-0.012
		(0.17)	(0.26)	(0.65)
Proportion of Non-Self Employed-Workers		-1.008	-1.211	-1.649
		(0.62)	(0.73)	(1.34)
Union Member			0.066*	
			(0.03)	
Democratic Governor			-0.003	
			(0.02)	
Right to Work Legislation			-0.023	
			(0.03)	
Log Wage Variance			-0.555	
			(0.32)	
Year Fixed-Effects?	No	No	No	Yes
State Fixed-Effects?	No	No	No	Yes

1, Additionally adjusted for individual union membership, state governor, Right to Work laws, and log wage variance
**, p<0.05; **, p<0.005; ***, p<0.001*

APPENDIX C: MARGINAL STRUCTURAL MODELS

Table A15. MSM Estimates of Periodized and Average Change in Union Membership on Agreement That Government Should Reduce Income Differences (Support for Income Redistribution)

	Model 1	*Model 2*
	Odds Ratio (Standard Error)	Odds Ratio (Standard Error)
Baseline Support for Redistribution	-0.079	-0.066
	(0.08)	(0.08)
Periodized (Ten Years) State Union Membership	0.634**	0.507*
	(0.19)	(0.21)
Average Change (Ten Years) in State Union Membership		-8.172*
		(3.73)
State (18+) Population	<-0.001*	<-0.001*
	(<0.01)	(<0.01)
Proportion of Young Workers	-0.900	-1.047
	(0.63)	(0.63)
Proportion of Female Workers	-0.287	-0.569
	(1.08)	(1.09)
Proportion of Non-White Workers	0.281*	0.309*
	(0.14)	(0.14)
Proportion of Non-Married Workers	0.684	0.707
	(0.42)	(0.42)
Proportion of Non-Postsecondary Workers	0.680**	0.609*
	(0.24)	(0.24)
Proportion of Non-Self-Employed Workers	-0.829	-0.824
	(0.55)	(0.56)
Year (Linear)	0.004	0.003
	(<0.01)	(<0.01)

Table A16. MSM Estimates of Periodized and Average Change in Union Membership on Agreement That Government Should Improve Living Standard for the Poor

	Model 1	*Model 2*

	Odds Ratio (Standard Error)	Odds Ratio (Standard Error)
Baseline Support for More Help for Poor	0.355*	0.352*
	(0.14)	(0.14)
Periodized (Ten Years) State Union Membership	1.040***	0.962**
	(0.29)	(0.32)
Average Change (Ten Years) in State Union Membership		-4.936
		(7.24)
State (18+) Population	<0.001	<0.001
	(<0.01)	(<0.01)
Proportion of Young Workers	0.870	0.784
	(1.08)	(1.07)
Proportion of Female Workers	-0.385	-0.399
	(1.84)	(1.84)
Proportion of Non-White Workers	0.313	0.331
	(0.23)	(0.24)
Proportion of Non-Married Workers	1.454	1.443
	(0.74)	(0.75)
Proportion of Non-Postsecondary Workers	1.291**	1.250**
	(0.40)	(0.42)
Proportion of Non-Self-Employed Workers	0.386	0.423
	(0.91)	(0.91)
Year (Linear)	0.008	0.008
	(<0.01)	(<0.01)
